# Factors Affecting Parent’s Perception on Air Quality—From the Individual to the Community Level

**DOI:** 10.3390/ijerph13050493

**Published:** 2016-05-12

**Authors:** Yulin Guo, Fengfeng Liu, Yuanan Lu, Zongfu Mao, Hanson Lu, Yanyan Wu, Yuanyuan Chu, Lichen Yu, Yisi Liu, Meng Ren, Na Li, Xi Chen, Hao Xiang

**Affiliations:** 1 Department of Epidemiology and Biostatistics, School of Public Health, Wuhan University, 115# Donghu Road, Wuhan 430071, China; sylvia-guo@foxmail.com (Y.G.); 2014283050041@whu.edu.cn (F.L.); zfmao@126.com (Z.M.); 2014203050033@whu.edu.cn (Y.C.); 2014203050031@whu.edu.cn (L.Y.); roselewis@sina.com (Y.L.); melodyren@126.com (M.R.); 2012302170047@whu.edu.cn (N.L.); aries_c_7@163.com (X.C.); 2 Global Health Institute, Wuhan University, 115# Donghu Road, Wuhan 430071, China; yuanan@hawaii.edu; 3 Environmental Health Laboratory, Department of Public Health Sciences, University of Hawaii at Mānoa, 1960 East-West Road, Honolulu, HI 96822, USA; yywu@hawaii.edu; 4 International Baccalaureate Diploma Program, Wuhan Foreign Languages School, Wan Song Yuan Road, Wuhan 430022, China; hansonlu_hl@hotmail.com

**Keywords:** perceived, air quality, factors, individual level, community level

## Abstract

The perception of air quality significantly affects the acceptance of the public of the government’s environmental policies. The aim of this research is to explore the relationship between the perception of the air quality of parents and scientific monitoring data and to analyze the factors that affect parents’ perceptions. Scientific data of air quality were obtained from Wuhan’s environmental condition reports. One thousand parents were investigated for their knowledge and perception of air quality. Scientific data show that the air quality of Wuhan follows an improving trend in general, while most participants believed that the air quality of Wuhan has deteriorated, which indicates a significant difference between public perception and reality. On the individual level, respondents with an age of 40 or above (40 or above: OR = 3.252; 95% CI: 1.170–9.040), a higher educational level (college and above: OR = 7.598; 95% CI: 2.244–25.732) or children with poor healthy conditions (poor: OR = 6.864; 95% CI: 2.212–21.302) have much more negative perception of air quality. On the community level, industrial facilities, vehicles and city construction have major effects on parents’ perception of air quality. Our investigation provides baseline information for environmental policy researchers and makers regarding the public’s perception and expectation of air quality and the benefits to the environmental policy completing and enforcing.

## 1. Introduction

Air pollution in China has become an increasingly severe problem in recent years. A report indicated that in 2013, only 4.1% cities among 74 monitored ones met official air pollution standards in terms of PM_2.5_, with an annual average PM_2.5_ value of 72 μg/m^3^; 14.9% of these cities met standards in terms of PM_10_, with an annual average value of 118 μg/m^3^ [1]. The association between air quality and respiratory diseases has been widely studied, and it is proven that air pollution is an important risk factor for respiratory diseases [2,3]. Children are the most vulnerable population to air pollutants, since they are at the stage of developing their pulmonary functions and physical growth. Moreover, the activity energy expenditure (AEE) values of children, especially preschoolers, are significantly higher than those of youths and adults [4]. Because children tend to be exposed to longer hours outdoors than adults [5], air pollution can be a serious health risk to growing children, not only by causing cough, asthma and other respiratory illness, but also inducing adverse pulmonary symptoms [6,7,8]. Therefore, the assessment of air quality and its health impact is particularly important.

Current information concerning urban air quality in China has been generally based on the measurements from scientific monitoring stations; little attention is paid to the general public regarding their feelings and subjective perceptions of air pollution. Actually, the evaluation of personal perceptions of air quality is an important aspect in life quality and health influence research. Research on air pollution perception, as well as health risk perception are crucial for understanding and predicting the consequences of environment contamination [9,10]; it is shown that compared to scientific air quality measurements, personal perception of air quality affects self-reported health conditions to a greater extent [11,12]. The National Environment Monitoring Program of Sweden, for example, has even included annoyance due to air pollution as one of its measures [13].

In many other countries, there were abundant research works about the relationship between perceived air quality and scientific measurements. A previous report indicated that air pollutant annoyance is strongly correlated with indoor concentrations of nitrogen dioxide and sulfur dioxide [14], while other researchers have shown that there is no significant relationship between perceived air quality and scientific measurements. A study conducted by Oglesby *et al.* in 2009 from Switzerland showed that annoyance at the community level is correlated with the local air quality, whereas annoyance at the individual level is only very weakly correlated with outdoor pollution levels [15]. Current evidence shows that air quality perception can be affected by many other factors, such as gender [13,15], age [16,17], residency status [18,19], level of education [13,18], health condition [13,17], family income [13,15] and household environment [18]. Besides individual-level factors, community-level factors also affect air quality perception. In developing countries, disparity in economic development, boosted by industrial advances, causes an uneven distribution of environment contamination [18]. It has been found that proximity to industrial areas affects the perception of air quality and the health risks of residents [17,20]. Residents that live in an area with heavy traffic also have a negative perception of air quality [18]. 

Wuhan, located in the middle reaches of the Yangtze River, is the largest city in central China. The city’s acceleration of industrialization and urbanization, sharp increasing number of automobiles and booming real estate industry pose great challenges to the air quality of Wuhan, which is the most heavily-polluted provincial capital city in southeast China [21]. As its economy develops, the population of Wuhan has rapidly increased by 21.6% since the last decade according to the sixth census of Wuhan in 2011. However, it is worth noting that the current study into the effects of air pollution on the health of children in Wuhan is generally focused on physiological aspects [22,23], but there is little literature referring to the perception of children’s parents on air pollution. Thus, the aim of this survey study is to explore the relationship between the perception of air quality of parents and the results of scientific measurements and to understand the factors that affect parents’ perception of air quality.

## 2. Methods 

### 2.1. Study Subjects

Through employing convenience sampling, two groups of parents whose children’s health conditions are disparate were chosen. The Children’s Hospital of Wuhan was chosen as a survey site, as it is the largest children’s hospital in Hubei Province. Another survey site was a community in Wuchang district, Wuhan City, the political, cultural and informational center of the whole province. Parents 18 years old or older with children between 1 and 12 years old were randomly surveyed, and they voluntarily took part in the study with the condition that they were fully informed of the aim and details of the research in advance. The sample size of the study was 1000.

### 2.2. Perceived Air Quality and Scientifically-Monitored Air Quality

The following questions were involved to examine parents’ perception of air quality: Compared to the air quality in Wuhan five years ago, how do you think of the air quality now? Candidate answer choices are: much better, better, same, worse, much worse and unsure (have less than 5 years’ experience in Wuhan, considering that there are some migrant workers, as well as parents coming from other cities for their children’s treatment). Consequently, the responses were dichotomized into two categories: better and worse. Responses of “unsure” were not included for regression analysis. Individual variables affecting parents’ perception of air quality concluded: basic information, health condition of child, concern level of air quality; community-level factors came from following question: which of the following do you think is/are the main cause(s) of air pollution in your city or town? Subjects were required to select the top 3 important sources from the above candidates.

**Figure 1:**
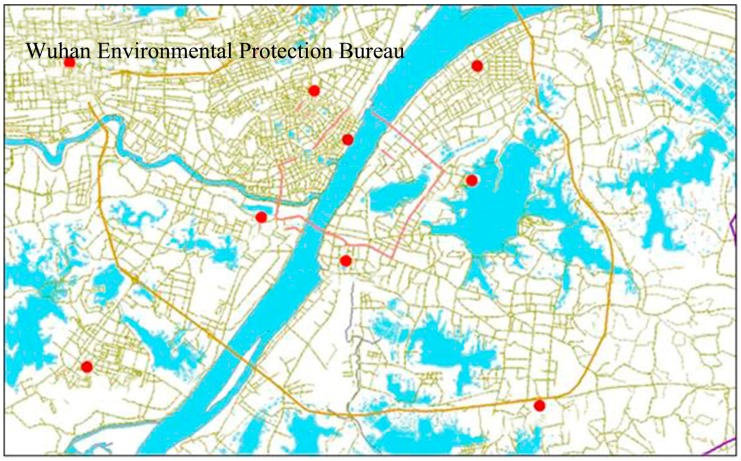
The map of air quality monitoring stations located in Wuhan City.

The data of the average concentrations of primary air pollutants and the annual excellent rate of air quality in Wuhan city were obtained from the official website of Wuhan Environmental Protection Bureau (Wuhan EPB) [24] and cover the period from 2010–2014. Wuhan EPB established 10 national-level monitoring stations for air quality. Among these 10 monitoring stations, Chenhu Qihao is 40 km away from the third ring road; it functions as the control sample reflecting the quality of background ambient air, and its data are not used to calculate the city-wide average concentration. The monitoring results of the other 9 monitoring stations are averaged into the city-wide air quality data. The distribution of the 9 monitoring stations is in Figure 1.

### 2.3. Quality Control

The questionnaires of this study were designed by experts from the Department of Public Health Sciences, University of Hawai’i. The survey study was conducted by the graduate students and faculty of the School of Public Health of Wuhan University with collaboration with the University of Hawaii. Before carrying out the survey, filling explanations were unified and participant students and faculty members were trained. Two preliminary surveys, each containing 30 questionnaires, were conducted in advance to ensure that questions on the questionnaire could be understood clearly by the respondents. During the survey, the quality control group would examine 10% of the responses randomly each day to make sure that there were no missed blanks, typographical errors, logical errors and to correct the errors as soon as they were found. 

### 2.4. Data Analysis

Epidata is employed to construct a database, and the double-blind method was used when the data were entered. Ineligible responses with key categorical data missing, such as gender and age, were eliminated. Statistical analysis was conducted by using SAS 9.1 (North Carolina State University, Raleigh, NC, USA). The univariable and multivariable logistic regression model was performed to evaluate the associations between variants and parents’ perception of air quality; variates included age, sex, residency status, education level, income, child health condition and degree of concern of air quality. OR and their 95% CI were calculated as estimates of the correlations, and a value of *p* < 0.05 was considered statistically significant in a two-tailed test.

### 2.5. Ethical Issues 

This study was approved by the Ethics Review Committee of Wuhan University (NO. 20140212) and the University of Hawaii (NO. 20140414) in accordance with the principles set forth by the Declaration of Helsinki of 1975, revised in 2008. Since the research is focused towards the general public, subjects voluntarily took part in the study with the condition that they were fully informed of the aim and details of the research in advance, and informed consent was obtained from all subjects. Furthermore, the survey was conducted anonymously; the personal information of subjects was not required; and the acquired data were only used for scientific analysis.

## 3. Results

### 3.1. General Information of Subjects

A total of 412 valid responses were collected from Wuhan Children’s Hospital and 453 from the community, with a valid returned rate of 82.4% and 90.6%, respectively. Table 1 shows the demographic data and related information of the subjects.

The gender compositions of the two groups are generally similar. The age of parents from the community is little higher than that of parents surveyed at Wuhan Children’s Hospital. The 30–39 years age group was the main part represented in both groups, with 45.6% of parents from the hospital group and 58.1% of parents from the community group. Thirty eight-point-eight percent of subjects surveyed at the hospital were in the age group of 18–29 years, while only 4.4% of respondents from the community group were in this age group. Correspondingly, 37.5% of respondents from the latter group and only 15.5% from the former group were 40 years and older. 

Most parents surveyed in the community resided in an urban area, with 399 people in total and a ratio of 88.5%. However, 412 respondents, namely, 44.4% of the hospital group, had an urban residency status and 55.6% had a rural one.

The educational background of parents from the community group was significantly higher than that from the hospital group. Seventy five-point-one percent of parents from the former group graduated from college, while only 27.7% of those from the latter group had such a degree of education. Sixty three-point-eight percent of parents surveyed at the hospital had a secondary school education level, and the ratio of elementary school education level is much lower.

Most subjects of the community group had an income of 50,000–150,000 Yuan, composing 64.0% of the group, whereas this ratio is 45.9% for the hospital group. Fifty percent of subjects from the hospital group had an income of 50,000 Yuan or below.

The health condition of the children from the community is significantly better than that of those from the hospital (*p* < 0.01). Seventy six-point-eight percent of respondents from the community reported that their child had a good condition, while this ratio is only 40.3% from the hospital. Correspondingly, 17.5% of respondents from the hospital reported poor child health, while only 1.8% of respondents from the community did so.

Among the subjects from the community group and the hospital group, 62.5% and 53.9% of subjects indicated a high concern degree for air quality, respectively.

### 3.2. Analysis of Scientific Measurements of Air Quality in Wuhan

#### 3.2.1. Annual Excellent Rate of Air Quality 

**Figure 2:**
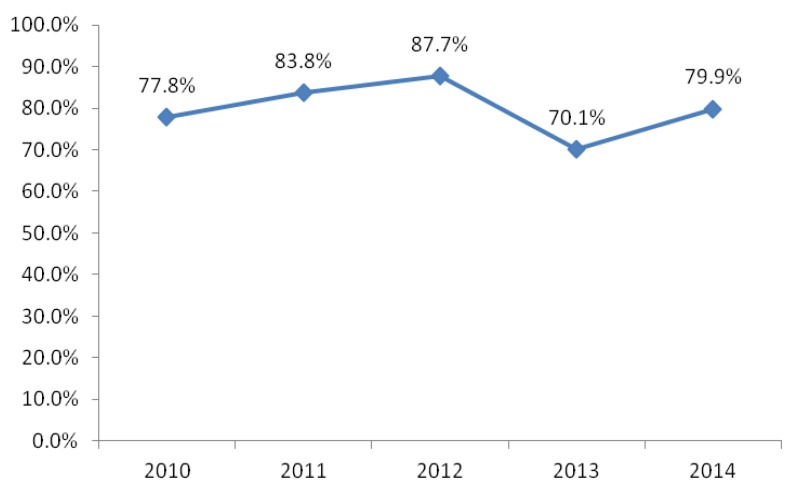
Variations of the annual excellent rate of air quality of Wuhan from 2010–2014.

The annual excellent rate of air quality of Wuhan from 2010–2014 was obtained from annual environmental condition reports. Figure 2 shows that the rate was 77.8% in 2010, and it is in a general rising tend from 2010–2014, except for the year 2013, with a decline to 70.1%; it reached to 79.9% in 2014.

#### 3.2.2. Variations of Average Air Pollutant Concentrations

**Figure 3:**
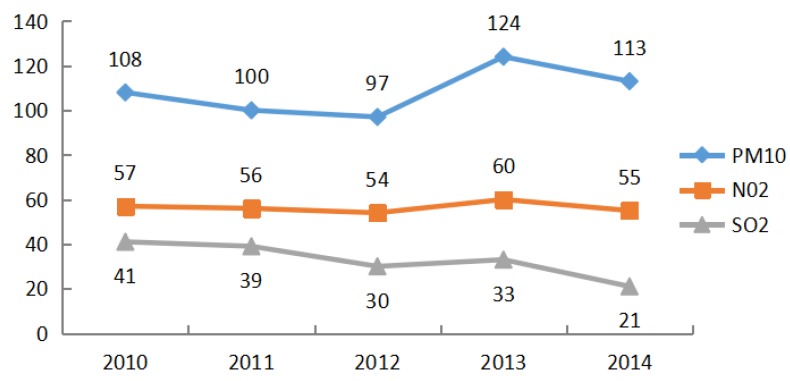
Variations of main air pollutant concentrations in Wuhan from 2010–2014.

NO_2_, SO_2_, PM_10_, CO, PM_2.5_ and O_3_ are the main pollutants monitored at the stations in Wuhan currently. CO, PM_2.5_ and O_3_ were newly listed as monitored pollutants in 2013. From 2010–2012, concentrations of NO_2_, SO_2_ and PM_10_ have been steadily declining, but suddenly increased and reached a peak value in 2013 and then declined sharply in 2014, as shown in Figure 3. The average concentration of PM_2.5_ was 94 μg/m^3^ and for PM_10_ was 124 μg/m^3^ in 2013, representing a value 1.7- and 1.8-times higher than the standard levels of PM_2.5_ and PM_10_, respectively. In 2014, the average PM_2.5_ concentration dropped to 82 μg/m^3^, and the PM_10_ average concentration declined to 113 μg/m^3^, which are still much higher (1.34- and 1.61-times) than the standard levels.

#### 3.2.3. Analysis of Parents’ Perception of Air Quality

The result of the perception of air quality between 2010 and 2014 shows that 84.3% of parents surveyed in the community and 74.2% of those surveyed at the hospital considered that the air quality has become worse. Therefore, most parents believe that the air quality in Wuhan has deteriorated in the past five years.

Table 2 shows logistic regression outcomes of air pollution perception of parents surveyed in the community at the individual level. Both univariate logistic regression and multilevel logistic regression analysis showed significant correlations between the perceptions of air quality of the respondents and their education level; the higher the education level of parents, the worse is the perception of parents of air quality.

Table 3 shows logistic regression outcomes of the air pollution perception of parents surveyed at the hospital at the individual level. Both univariate logistic regression and multilevel logistic regression analysis revealed that subjects’ perceptions of air quality are significantly correlated to their age, resident area, education level and the health condition of their children. Subjects with an age of 40 years or above, urban resident area, college or above education level and poor child health condition have a worse perceived air quality. In addition, univariate logistic regression analysis shows that there are significant correlations between the perceptions of air quality of the respondents and their resident area; however, in the multilevel logistic regression analysis, there is no association between them.

### 3.3. The Effect of Community-Level Factors on Air Quality Perception

Parents’ perception of air quality was not only affected by individual characteristics, but also by community environments. During the survey, the main air pollution sources were analyzed and showed that automobiles, industrial facilities and city construction were chosen by parents as the most important sources of pollution, with ratios of 69.4%, 55.7% and 53.3%, respectively. The result is in agreement with official reports basically. According to a recent primary research on the composition of pollutant particulate sources, external sources make up 20%–30% of the total calculation, and endogenous sources are the main cause of pollution. Such internal sources include industrial and coal emissions, composing about 40% of all endogenous contaminants, dust pollution, composing approximately 25%, and vehicle emissions, comprising about 20% [25]. We converted these three factors into quantifiable indicators: the number of motor vehicles, GDP growth and urban construction investment in Wuhan; and then, we searched related information from 2010–2014 and drew out the trends of community-level factors.

**Figure 4:**
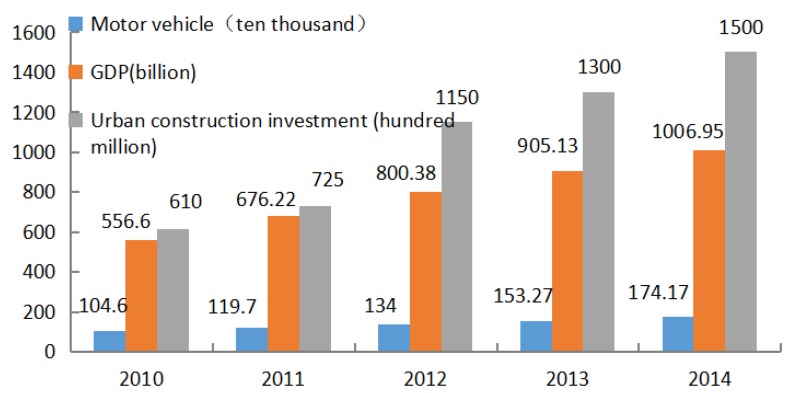
Trends of community-level factors.

Figure 4 shows the trends of community-level factors. According to data released from the City Construction Committee of Wuhan, city construction investments have a steady fast-paced increase in the past five years, with a total of 61 billion Yuan in 2010 to 150 billion Yuan in 2014. Data released by the Statistics Bureau of Wuhan show that the GDP of Wuhan has also followed with a rising trend, from 556.59 billion Yuan in 2010 to 1.0069 trillion Yuan in 2014. Data from the transportation authority of the city indicate that the amount of automobiles in Wuhan has increased from 1.05 million in 2010 to 1.74 million in 2014.

## 4. Discussion

Our study is completed with two types of parent populations: one group is the children’s parents from a community in Wuchang, and the other is the parents with sick children in Wuhan Children’s Hospital. The two groups have significant discrepancies in resident area, showing that over 88% (453) of parents surveyed in the community lived in urban areas, while only 45% of the respondents of the hospital group lived in urban areas. Moreover, referring to the cognition of air quality in Wuhan, 27.4% of the participant parents of the hospital group responded with the choice “unknown” (resided in Wuhan for less than five years). This could largely be due to the majority of the parents from the hospital group not being local residents (Wuhan), and they were mobile people living in the city for less than five years, or they lived in a region close to Wuhan and came to the hospital for their children’s sickness. The mobile population of Wuhan has grown significantly in recent years due to the rapid economic development. According to a survey conducted by the Chinese Academy of Sciences in 2013, the floating population of Wuhan reached 2.87 million, of which the majority comes from rural areas [26]. 

According to the environmental reports of Wuhan, it can be concluded that the air quality of Wuhan in 2014 remained the same as it was in 2010, though it experienced a sharp decline in 2013, indicating that the air quality control measurement has been effective. In fact, the sharp decline of air quality in 2013 might be due to numerous nation-wide extreme climate conditions. During the year, record-breaking conditions of hazy weather occurred, which was unprecedentedly frequent and pervasive, appearing at a peak value for the past 52 years [1]. In addition, Zhang and co-workers described that the climate conditions in 2013 were greatly different from other years with relatively low humidity and precipitation, more static weather, and the atmosphere was stagnant, thereby weakening the possibility for pollutants to dissipate [27]. However, parents considered the air quality of Wuhan to be in a worse condition in 2014 compared to 2010. Factors from both individual and community aspects were examined for their effect on their perception, to explain the distinction between the perception and reality. 

Comparing the different age groups, we found that the older ones have a more negative perception. This result is inconsistent with the study of Howel *et al.*, which stated that old-age respondents are unlikely to give current air quality a low rating, since they had a worse environmental experience during former decades [16]. This difference may reflect the difference for the age defined by the two studies. In Howel *et al.*’s survey, 75 years or above is classified into the older age group compared to 40 years or above, which was considered to be old in this study. We infer that the middle-aged (40 years or above) parents were more likely to emphasize health and safety compared to young groups. Moreover, research conducted by Kim *et al.* in 2012 from Korea showed that subjects at a young age tend to have more negative perceptions of air quality [28]. This population group mainly consists of students, who were more concerned about environmental issues [6].

Univariate logistic regression analysis of resident area shows that parents living in urban areas have a more negative perception of air quality. Literature relating to the effect of resident area on perception of air quality is limited so far. A study conducted in Shanghai in 2013 showed that perception of air quality differed significantly among residents in different districts of the city. The more urbanized the area in which subjects reside, the more serious they believed the smog problem was [19]. Such a disparity is related to heavy vehicle emissions and the density of buildings in city centers. A study conducted by Brody *et al.* [17] from Houston and Dallas revealed that compared to citizens in urban areas, subjects who lived in rural areas perceived air quality more accurately. However, in the multilevel logistic regression analysis, there is no correlation in resident area, so we consider it as a confounding factor. It has been well proven that urban and rural residents are unequal in terms of their income and education level. In the central region of China, fertility patterns of urban residents also differ from those of rural residents; the former tends to give birth at a later age (>30 years old) [29,30].

This study also demonstrated that parents with high education have a more negative perception of air quality. This may be due to education having an effect on economic, vocational and social status to different extents. This result is consistent with a previous report showing that the cognition of air quality of citizens is related to their education level [31]. In addition, Badland *et al.* [32] also found that compared to population groups with low education levels, those who receive higher education tend to have a better understanding that air pollution can adversely affect their health and are therefore more concerned about environmental issues. Correspondingly, their perception of air quality is worse. 

This study chose a hospital as one site and found that parents whose children have worse health conditions view air quality more negatively. Abundant literature has proven that air pollution exacerbates children’s respiratory symptoms [33,34]. Yazdanparast *et al.* [35] has shown that parents with asthmatic children will tend to be more aware of the hazards of air pollution. These studies provide support for our results.

Personal characteristics of subjects from the two groups are significantly disparate in general. Compared to subjects surveyed at the hospital, community parents are older in age and have a higher level in education and family income. However, though the individual level variables of the two groups are different, their concern and awareness of air pollution and perception of air quality are strikingly consistent. Such a trend indicates that air pollution has high awareness among the general public.

At the community level, we analyzed the three main sources of air pollution in Wuhan, namely city construction, industrial facilities and vehicle emissions. By looking at average concentrations of major air pollutants of Wuhan from 2010–2014, we showed that PM_10_ and PM_2.5_ had dramatically exceeded the level of health standards since 2013, representing the first two primary air pollutants. Further analysis showed that the main source of PM_10_ is dust from soil and roads, accounting for about 50% [36]. In recent years, investment in city construction in Wuhan has greatly increased; there were over 11,000 road construction, civil projects and real estate construction sites in Wuhan in 2015, causing much dust pollution. Previous research showed that subjects are highly aware of visible pollutants, such as dust [37], which led them to have a worse perception of air quality. 

A survey conducted in six major cities in China from November 2013–February 2014 (89 days in total) indicated that among these cities, Wuhan has the highest average concentration of PM_2.5_, reaching a value of 146.15 μg/m^3^, which indicates 1.82-times over the standard level. The survey has also shown that the industrial proportion and the speed of comprehensive development of the city has contributed to the increased PM_2.5_ concentrations greatly [38]. From 2010–2014, the GDP of Wuhan increased steadily and reached 1.0069 trillion Yuan in 2014. The chemical industry was identified to take the largest proportion of heavy industry and consumes 61.4% of the total energy in whole city. It is said that the essence of air pollution in Wuhan is PM_2.5_ [39]. Studies reveal an inverse causal relationship between atmosphere visibility and PM_2.5_ concentration [40,41]. Since 2012, PM_2.5_ has been listed as one of the monitored pollutants by the newly-revised Ambient Air Quality Standards [10] and has widely attracted media and public attention. “Haze” became the keyword in 2013 due to the severe and prevalent smoggy weather. We may therefore deduce that deteriorated weather caused by industrial development and the exhaustive reports by the media on PM_2.5_ caused children’s parents to have a more negative impression of air quality.

With the improvement of living standards, the amount of automobiles in Wuhan has increased from 1.046 million in 2010 to 1.742 million in 2014. Nitrogen oxide emitted from automobiles contributes to 31.1% of the total amount, but in fact, its concentration has been following a general decreasing trend, thanks to effective air pollution control measures, such as outlawing yellow-labeled vehicles (vehicles with high emission levels). However, residents of Wuhan still tend to attribute air pollution primarily to automobiles today, and this tendency may be caused by the traffic congestion due to the increase in the amount of vehicles. The literature has shown that residents living near heavy traffic have a more negative impression of air quality [13].

## 5. Conclusions

Our research shows that in general, subjects have a negative impression of air quality, which differs greatly from scientific measurements. Through statistical analysis, we found that at the individual level, perception is significantly correlated with resident area, age, education level and the health condition of their children. At the community level, public perception is affected by automobiles, industrial facilities and city construction. Although there is a complex variety of factors, the subjects from both groups are paying great attention to air pollution. In addition, their perception of air quality is an important factor for their acceptance of environment policies set by the government. In fact, the local government has made great efforts to improve the air quality in recent years and brought results, but in this survey, the public perceived that air quality has been deteriorating continuously*.* Some Chinese scholars have already proposed that the survey and analysis of the air quality satisfactory rate in cities can be a supplement to scientific monitoring in terms of evaluating urban air quality and the effectiveness of government control measurement [42]. Our findings may help policy makers to ascertain a clearer understanding of the relationship between the perception of the general public towards air pollution and scientific measurements and assist them in the dissemination of necessary information to the general public for the better feasibility of their policies.

### Limitations of Our Research

In our research, only two survey sites were selected, and the research was not conducted on the whole population of Wuhan; therefore, the sample range should be enlarged. Regarding the analysis of air quality in the recent five years of Wuhan, PM_2.5_ concentrations, an important measurement in evaluating air quality, were only used in 2013 and 2014, due to the lack of data prior to 2013. In the hospital, we mainly carried out the survey in the department of respiratory diseases; we made sure that the majority of children were suffering from respiratory diseases, while we did not collect the children’s diseases. If information on the specific category and extent of the disease of children with a poor health condition were collected, in the analysis of the health condition of children, the accuracy of the results of our research may be improved. In future research, these problems may be improved, and more individual- and community-level factors may be included.

## Figures and Tables

**Table 1:** General data of the two population groups.

Item	Community (*n* = 453)		Hospital (*n* = 412)		*p-*Value
	*N*	Percentage	*N*	Percentage	
**Gender**					
Male	181	40.0	187	45.4	0.11
Female	272	60.0	225	54.6	
**Age Group (years)**					
18–29	20	4.4	160	38.8	<0.01
30–39	263	58.1	188	45.6	
40 and above	170	37.5	64	15.5	
**Residency Status**					
Rural	52	11.5	229	55.6	<0.01
Urban	401	88.5	183	44.4	
**Education Level**					
Elementary school	28	6.2	35	8.5	<0.01
Secondary school	85	18.8	263	63.8	
College and above	340	75.1	114	27.7	
**Annual Family Income (Yuan)**					
<50,000	80	20.4	206	50.0	<0.01
50,000–15,000	251	64.0	189	45.9	
>15,000	61	15.6	17	4.1	
**Health Condition of Child**					
Good	348	76.8	166	40.3	<0.01
Moderate	97	21.4	174	42.2	
Poor	8	1.8	72	17.5	
**Degree of Concern of Air Quality**					
Rather concerned	17	3.7	16	3.9	<0.05
Concerned	153	33.8	174	42.2	
Highly concerned	283	62.5	222	53.9	

**Table 2:** Logistic regression analysis (OR and 95% CI) for air quality perception: at the individual level in the community group.

Item	Total *N* (%)	Air Quality Perception		Crude OR (95% CI)	Adjusted OR (95% CI)
		Better (*N*/%)	Worse (*N*/%)		
		69 (15.7)	371 (84.3)		
Gender					
Male	178 (40.4)	31 (44.9)	147 (39.6)	Ref.	Ref.
Female	262 (59.6)	38 (55.1)	224 (60.4)	1.243 (0.741, 2.087)	0.947 (0.539, 1.666)
Age Group (years)					
18–29	16 (3.6)	2 (2.9)	14 (3.8)	Ref.	Ref.
30–39	256 (58.2)	32 (46.4)	224 (60.4)	1.000 (0.217, 4.605)	1.384 (0.280, 6.834)
40 and above	168 (38.2)	35 (50.7)	133 (35.8)	0.543 (0.118, 2.501)	0.785 (0.158, 3.889)
Residential Status					
Rural	52 (11.8)	9 (13.0)	43 (11.6)	Ref.	Ref.
Urban	388 (88.2)	60 (87.0)	328 (88.4)	1.144 (0.530, 2.470)	1.007 (0.529, 1.918)
Education Level					
Elementary school	28 (6.4)	11 (15.9)	17 (4.6)	Ref.	Ref.
Secondary school	83 (18.9)	12 (17.4)	71 (19.1)	3.828 (1.445, 10.144)	4.164 (1.491, 11.628) **
College and above	329 (74.8)	46 (66.7)	283 (76.3)	3.981 (1.753, 9.038) **	4.034 (1.610, 10.110) **
Child Health Condition					
Good	336 (76.4)	51 (73.9)	285 (76.8)	Ref.	Ref.
Moderate	96 (21.8)	16 (23.2)	80 (21.6)	0.895 (0.484, 1.653)	0.956 (0.503, 1.814)
Poor	8 (1.8)	2 (2.9)	6 (1.6)	0.537 (0.105, 2.734)	0.570 (0.106, 3.053)
Annual Family Income (Yuan)					
<50,000	79 (18.0)	15 (21.7)	64 (17.3)	Ref.	Ref.
50,000–150,000	242 (55.0)	39 (56.5)	203 (54.7)	1.220 (0.632, 2.357)	1.207 (0.609, 2.393)
>150,000	61 (13.9)	7 (10.1)	54 (14.5)	1.808 (0.687, 4.758 )	1.523 (0.557, 4.162)
Unknown	58 (13.1)	8 (11.6)	50 (13.5)	1.465 (0.575, 3.729 )	1.759 (0.642, 4.817)
Degree of Concern of Air Quality					
Rather concerned	16 (3.6)	2 (2.9)	14 (3.8)	Ref.	Ref.
Concerned	146 (33.2)	31 (44.9)	115 (31.0)	0.530 (0.114, 2.457)	0.481 (0.098, 2.352)
Highly concerned	278 (63.2)	36 (52.2)	242 (65.2)	0.960 (0.210, 4.401)	0.838 (0.172, 4.076)

Notes: OR = odds ratio; CI = confidence interval. ** *p* < 0.01.

**Table 3:** Logistic regression analysis (OR and 95% CI) for air quality perception: at the individual level of the hospital group.

Item	Total *N* (%)	Air Quality Perception		Crude OR (95% CI)	Adjusted OR (95% CI)
		Better (*N*/%)	Worse (*N*/%)		
		77 (25.8)	222 (74.2)		
Gender					
Male	133 (44.5)	36 (46.8)	97 (43.7)	Ref.	Ref.
Female	166 (55.5)	41 (53.2)	125 (56.3)	1.132 (0.672, 1.904)	1.230 (0.685, 2.208)
Age Group (years)					
18–29	109 (36.5)	39 (50.6)	70 (31.5)	Ref.	Ref.
30–39	142 (47.5)	31 (40.3)	111 (50.0)	1.995 (1.141, 3.487) *	1.922 (1.024, 3.605) *
40 and above	48 (16.0)	7 (9.1)	41 (18.5)	3.263 (1.337, 7.963) **	3.252 (1.170, 9.040) *
Residency Status					
Rural	144 (48.2)	47 (61.0)	97 (43.7)	Ref.	Ref.
Urban	155 (51.8)	30 (39.0)	125 (56.3)	2.019 (1.189, 3.427) **	1.007 (0.529, 1.918)
Education Level					
Elementary school	21 (7.0)	10 (13.0)	11 (5.0)	Ref.	Ref.
Secondary school	180 (60.2)	57 (74.0)	123 (55.4)	1.962 (0.788, 4.884)	1.879 (0.678, 5.210)
College and above	98 (32.8)	10 (13.0)	88 (39.6)	7.997 (2.722, 23.489) **	7.598 (2.244, 25.732) **
Child Health Condition					
Good	117 (39.1)	42 (54.5)	75 (33.8)	Ref.	Ref.
Moderate	130 (43.5)	31 (40.3)	99 (44.6)	1.788 (1.029, 3.108) *	1.779 (0.978, 3.237)
Poor	52 (17.4)	4 (5.2)	48 (21.6)	6.720 (2.265, 19.941) **	6.864 (2.212, 21.302) **
Annual Family Income (Yuan)					
<50,000	134 (44.8)	42 (54.5)	92 (41.4)	Ref.	Ref.
50,000–150,000	150 (50.2)	33 (42.9)	117 (52.7)	1.619 (0.951, 2.754)	1.180 (0.655, 2.127)
>150,000	15 (5.0)	2 (2.6)	13 (5.9)	2.967 (0.641, 13.742)	1.699 (0.334, 8.645)
Degree of Concern of Air Quality					
Rather concerned	10 (3.3)	4 (5.2)	6 (2.7)	Ref.	Ref.
Concerned	113 (37.8)	29 (37.7)	84 (37.8)	1.932 (0.509, 7.333)	1.071 (0.254, 4.517)
Highly concerned	176 (58.9)	44 (57.1)	132 (59.5)	2.001 (0.540, 7.420)	0.956 (0.233, 3.921)

Notes: OR = odds ratio; CI = confidence interval. * *p* < 0.05; ** *p* < 0.01.
